# Acute Infusion Pain Reaction Due to Anti-PD-1 Antibodies for the Treatment of Cutaneous Squamous Cell Carcinoma in Recessive Dystrophic Epidermolysis Bullosa: A Case Report and Review of the Literature

**DOI:** 10.1155/crdm/4558623

**Published:** 2025-02-26

**Authors:** Vanessa Tran, Susan J. Robertson, Jamie Young, Malcolm Hogg, Alesha A. Thai, Vanessa Morgan

**Affiliations:** ^1^Department of Dermatology, The Royal Melbourne Hospital, Parkville, Victoria, Australia; ^2^Department of Medicine, University of Melbourne, Parkville, Victoria, Australia; ^3^Department of Dermatology, The Royal Children's Hospital, Parkville, Victoria, Australia; ^4^Murdoch Children's Research Institute, Parkville, Victoria, Australia; ^5^Department of Anaesthesia and Pain Medicine, Peter MacCallum Cancer Centre, Melbourne, Victoria, Australia; ^6^Department of Critical Care, University of Melbourne, Parkville, Victoria, Australia; ^7^Department of Anaesthesia and Pain Management, Royal Melbourne Hospital, Parkville, Victoria, Australia; ^8^Department of Oncology, Peter MacCallum Cancer Centre, Melbourne, Victoria, Australia; ^9^Sir Peter MacCallum Department of Oncology, University of Melbourne, Parkville, Victoria, Australia

**Keywords:** cemiplimab, epidermolysis bullosa, immune checkpoint inhibitor, pain, squamous cell carcinoma

## Abstract

Recessive dystrophic epidermolysis bullosa (RDEB) belongs to a rare group of inherited dermatoses, which are characterised by mucosal and cutaneous fragility. Cutaneous squamous cell carcinoma (CSCC) is a common complication of RDEB. In the severe subtype of RDEB (RDEB-S), CSCC is observed in 90% of the patients by 55 years. CSCC in patients with RDEB follows an aggressive course with the median survival rate of 2.4 years. We report the case of a 51-year-old female with RDEB with recurrent aggressive CSCC of the right lateral-back. She was commenced on cemiplimab, an anti-programmed death receptor-1 (PD-1) antibody, for the management of unresectable locally advanced CSCC; however, she experienced a severe infusion reaction, manifested as back pain, requiring treatment cessation. Despite three incomplete doses, the patient demonstrated a marked response with significant regression of her tumours. Therefore, further treatment was pursued. She was successfully administered cemiplimab under intravenous sedation. This was later complicated by immune-related colitis, necessitating treatment cessation. The patient was transitioned to best supportive care. The patient required inpatient admission for end-of-life care due to her complex analgesia requirements. This case report explores the pathophysiological mechanisms of pain in RDEB and anti-PD-1 antibody therapy and highlights the unique challenges of pain management in RDEB patients.

## 1. Introduction

Recessive dystrophic epidermolysis bullosa (RDEB) is a rare inherited disorder of skin and mucosal fragility. This genodermatosis is characterised by pathogenic variants of the *COL7A1* gene, which encodes type VII collagen. Deficiency of collagen type VII in RDEB results in recurrent blisters, erosions and ulcerations following only minimal trauma. In the severe form of RDEB (RDEB-S), significant extracutaneous complications can develop, which may be life-limiting [1]. Cutaneous squamous cell carcinoma (CSCC) in RDEB-S typically demonstrates an aggressive course [2, 3]. It is predicted that over 90.1% of the patients with RDEB will be diagnosed with CSCC by the age of 55 with a mortality rate of 87.3% by the age of 45 [4]. The median survival of patients with RDEB-S following initial CSCC diagnosis is 2.4 years [3]. While surgical management is the standard treatment of choice for CSCC, complete resection with adequate margins is not always feasible [5]. The use of immune checkpoint inhibitors (ICIs), such as cemiplimab, is established as the standard of care in patients with unresectable or metastatic CSCC but data regarding its efficacy in patients with RDEB and CSCC is limited [6, 7].

Herein, we describe the case of a patient with RDEB-S, who underwent treatment of metastatic CSCC with cemiplimab. Her treatment course was complicated by infusion reactions manifesting as severe back pain, eventually requiring intravenous sedation to successfully administer a complete dose. Our case is unique as it describes the multimodal management of pain for the administration of systemic therapy in a patient with RDEB-S and complex chronic pain.

## 2. Case Report

**Figure 1:**
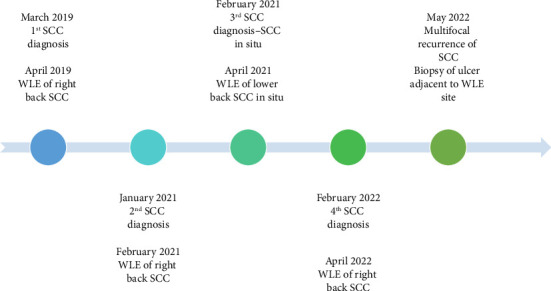
Timeline of squamous cell carcinoma diagnoses. SCC = squamous cell carcinoma. WLE = wide local excision.

We present a 51-year-old female with RDEB-S, confirmed by genetic testing (heterozygous *COL7A1* variants), with manifestations of chronic wounds, ectropion with exposure keratitis, corneal scarring, osteopaenia and oesophageal strictures, requiring multiple dilations and nutritional support via percutaneous endoscopic gastrostomy tube. The patient also suffered chronic pain requiring moderate-dose opioid analgesia at the baseline. She presented with multifocal recurrent CSCC of the right back. She had undergone two previous wide local excisions (WLEs) at the same anatomical site, with well-differentiated CSCC (April 2019) followed by moderately differentiated CSCC (February 2021), both successfully completely excised with > 1 cm margins. A separate SCC in situ (April 2021) on the lower back was also successfully treated by WLE. The patient presented in February 2022 with multiple new foci on the right back, with biopsies proving CSCC. She again underwent WLE, with pathology demonstrating poorly differentiated CSCC with margins of 2.5 mm (Figure 1). The clinical and pathological impression was that this reflected multifocal recurrence without evidence of cutaneous metastases.

Only 2 months after the last resection, the patient developed multiple suspicious lesions and biopsy confirmed multifocal recurrence of CSCC. Due to the progression of disease, it was felt that surgery would unlikely provide a long-term cure. The patient, herself, also declined further operative management. She was referred to a medical oncologist and granted compassionate access to cemiplimab for management of unresectable CSCC.

The patient was scheduled to receive 350 mg cemiplimab in 64 mL normal saline. During infusion of the first cycle, the patient developed severe back pain, necessitating interruption of the infusion. Hydrocortisone and paracetamol were administered and an attempt was made to restart the infusion at half the rate. This, however, was again terminated due to the recrudescence of symptoms. A rechallenge of cemiplimab was attempted one week later with hydrocortisone, promethazine and paracetamol premedication. The patient again experienced severe back pain despite intravenous morphine 15 mg, and the infusion was aborted after the administration of 4.8 mL out of a total 64 mL of cemiplimab.

**Figure 2:**
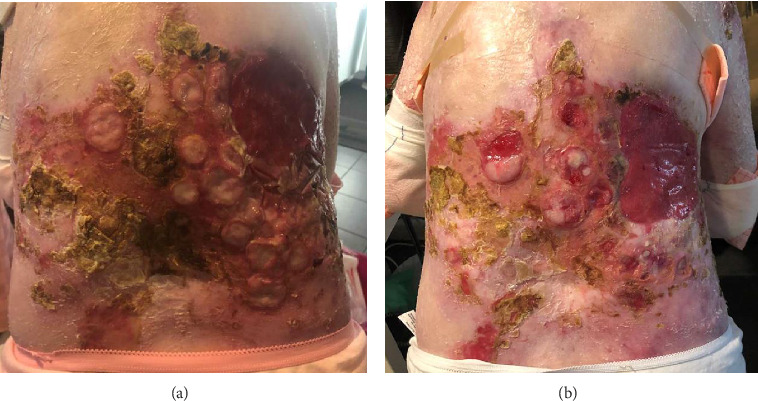
Images of the back demonstrating multifocal squamous cell carcinoma. (a) Prior to cemiplimab in June 2022 and (b) after attempted cycle 2 in August 2022. Objective reduction in the size of the multifocal local recurrent cutaneous SCCs was observed and the patient also reported reduced pain symptoms from the lesions.

Despite only receiving a small volume of cemiplimab, the patient noted marked improvement in baseline pain and appearance of the CSCC lesions (Figure 2). Given the positive response, a decision was made to pursue another cycle of treatment with additional analgesia. The patient was first admitted to the medical oncology ward for an intravenous ketamine infusion for opioid desensitisation. This infusion was incrementally increased from 4 mg per hour to 24 mg per hour over 4 days. The patient was then taken to theatre and received the full cemiplimab infusion under intravenous propofol sedation. A premedication regimen of hydrocortisone, promethazine and paracetamol was also administered in accordance with the NCCN guidelines for the management of immunotherapy-related toxicities [8]. The patient tolerated the infusion under sedation, although suffered a short flare of neck and back pain postinfusion, which was adequately treated with increased analgesia.

Four weeks following this infusion, the patient developed immune-related colitis, requiring admission to hospital for intravenous methylprednisolone. Cemiplimab was discontinued and the patient was referred for palliative radiotherapy for symptomatic management of the CSCC at her back and progressive inguinal lymphadenopathy. A dose of 20 Gray over 5 fractions (20 Gy/5#) was successfully delivered to the lower back. Radiotherapy to the groin (20 Gy/5#) was also planned but was terminated early after one fraction due to radiation-induced diarrhoea.

Due to uncontrolled symptoms in the community, the patient was admitted to hospital for intravenous multimodal analgesia and inpatient end of life care. She passed away in the hospital on the Palliative Care ward, approximately 4 years after the initial diagnosis of CSCC.

## 3. Discussion

This case describes the manifestation of acute pain in a patient with RDEB-S receiving an ICI for the management of unresectable CSCC. Importantly, this individual suffered from chronic pain due to chronic ulcerations and wounds. Her disease course was complicated by fear of the unknown and loss of control. Our case generates several unique discussion points pertaining to the mechanisms and management of pain in patients with RDEB. In this discussion, we explore the implication of cemiplimab in pain pathways and propose a possible mechanism for infusion pain reactions. In addition, we emphasise the need for multimodal management in the broader context of a patient with complex chronic pain and a progressive life-limiting condition.

Cemiplimab binds to programmed death receptor-1 (PD-1), thereby blocking the PD-1/programmed death-ligand 1 (PD-L1) pathway. It has demonstrated impressive and durable responses in locally advanced and metastatic CSCC [6, 7]. There is emerging evidence of its efficacy for patients with unresectable or metastatic CSCC with RDEB [3, 8, 9]. Infusion reactions manifested as musculoskeletal pain is recognised in ICIs, including cemiplimab [6, 7]. In addition, severe back pain has been reported as a nonimmune adverse event in ICIs for treatment of other malignancies [10]. The mechanism by which this occurs is not clear in the literature. However, the relationship between PD-1/PD-L1 and pain has been described.

The PD-1/PD-L1 pathway has been implicated in acute and chronic pain through the modulation of neuroinflammation. PD-L1 is widely expressed on neuronal tissue and skin and has been shown to suppress pain signalling through interaction with its receptor, PD-1. In peripheral nerve damage, there is upregulation of PD-L1 expression, suggesting its role in the suppression of pain after injury [11]. In addition, blockade or deficiency of PD-1/PD-L1 has been shown to induce pain and allodynia in mice studies [12]. In clinical practice, however, the role of PD-1/PD-L1 inhibitors in the propagation of pain has not been explored.

In RDEB, there are complex bio–psycho–social mechanisms by which significant, refractory pain in patients may occur. The burden of pain is highest in RDEB patients compared with other EB subtypes [13, 14]. Repeated blistering and injury to the skin in RDEB has been shown to damage small sensory fibres resulting in neuropathic pain [15]. Chronic neuroinflammation results in sustained changes in nociceptor function, manifesting in hyperalgesia and allodynia [16]. In chronic pain, this is compounded by the development of opioid-induced hyperalgesia and resultant opioid tolerance [17]. Pain management is, therefore, complicated by the combination of amplified pain and decreased efficacy of opioid analgesia. In addition, chronic pain sufferers, such as those with RDEB often develop a negative emotional response towards pain. Such rumination or anticipation of pain can detrimentally impact one's ability to respond to pain management strategies [18, 19]. In our case, the pain was perpetuated by the fear of pain recurrence and compounded by previous experiences with uncontrolled pain. This was further compounded by the fear of the unknown and a loss of control in a young individual.

The management of pain and anaesthesia in RDEB patients presents unique challenges. In addition to increased opioid requirements, RDEB patients present an airway risk due to mucosal fragility and comorbid oral and oesophageal strictures and thus procedural sedation carries heightened risk [20]. The use of intravenous ketamine and propofol infusions has previously been described for dressing changes and whirlpool baths in paediatric RDEB patients [21]. The addition of ketamine to propofol during procedural sedation has demonstrated favourable effects on haemodynamics and respiration, improved sedation and reduced narcotic requirements [22].

This case describes the unique management of an acute infusion-related pain reaction in a patient with RDEB-S. We propose that patients with RDEB are at higher risk of severe pain reactions due to multiple mechanisms driven by chronic pain and inflammation. Clinicians must be aware of the complexities in pain management for patients with RDEB. In this patient cohort, where CSCC historically displays an aggressive course and confers poor prognosis, we highlight the need for a multidisciplinary approach and careful consideration of the patient's comorbidities for a tailored treatment plan.

## Data Availability

The data that support the findings of this study are available from the corresponding author upon reasonable request.
